# Mind wandering and motor control: off-task thinking disrupts the online adjustment of behavior

**DOI:** 10.3389/fnhum.2012.00329

**Published:** 2012-12-14

**Authors:** Julia W. Y. Kam, Elizabeth Dao, Patricia Blinn, Olav E. Krigolson, Lara A. Boyd, Todd C. Handy

**Affiliations:** ^1^Attentional Neuroscience Lab, Department of Psychology, University of British ColumbiaVancouver, BC, Canada; ^2^Aging, Mobility and Cognitive Neuroscience Laboratory, Department of Physical Therapy, University of British ColumbiaVancouver, BC, Canada; ^3^Neuroeconomics Laboratory, Department of Psychology and Neuroscience, Dalhousie UniversityHalifax, NS, Canada; ^4^Brain Behavior Laboratory, Department of Physical Therapy, University of British ColumbiaVancouver, BC, Canada

**Keywords:** mind wandering, experience sampling, motor control, visuomotor tracking task, fERN, performance monitoring, time-estimation

## Abstract

Mind wandering episodes have been construed as periods of “stimulus-independent” thought, where our minds are decoupled from the external sensory environment. In two experiments, we used behavioral and event-related potential (ERP) measures to determine whether mind wandering episodes can also be considered as periods of “response-independent” thought, with our minds disengaged from adjusting our behavioral outputs. In the first experiment, participants performed a motor tracking task and were occasionally prompted to report whether their attention was “on-task” or “mind wandering.” We found greater tracking error in periods prior to mind wandering vs. on-task reports. To ascertain whether this finding was due to attenuation in visual perception *per se* vs. a disruptive effect of mind wandering on performance monitoring, we conducted a second experiment in which participants completed a time-estimation task. They were given feedback on the accuracy of their estimations while we recorded their EEG, and were also occasionally asked to report their attention state. We found that the sensitivity of behavior and the P3 ERP component to feedback signals were significantly reduced just prior to mind wandering vs. on-task attentional reports. Moreover, these effects co-occurred with decreases in the error-related negativity elicited by feedback signals (fERN), a direct measure of behavioral feedback assessment in cortex. Our findings suggest that the functional consequences of mind wandering are not limited to just the processing of incoming stimulation *per se*, but extend as well to the control and adjustment of behavior.

## Introduction

Mind wandering, or those transient periods of time during which our attention momentarily drifts away from our on-going task and perceptual milieu, is fundamental to human neurocognitive function. In terms of neural architecture, mind wandering episodes have been strongly associated with activation of the brain's default mode network (e.g., Mason et al., 2007; Christoff et al., 2009; Kirschner et al., 2012), while in terms of cognitive processes, mind wandering has been tied to fluctuations in executive control (e.g., McVay and Kane, 2009, 2012). Such findings have supported the hypothesis that regular oscillations in the depth of our neurocognitive engagement with the external environment is normative to healthy human brain function (e.g., Smallwood and Schooler, 2006; Schooler et al., 2011; Smallwood, in press), and that a variety of clinical and sub-clinical cognitive pathologies may be linked to altered patterns of mind wandering (e.g., Shaw and Giambra, 1993; Helton, 2009; Smallwood et al., 2009; Killingsworth and Gilbert, 2010; Elua et al., 2012).

Given that mind wandering is central to our neurocognitive make-up, there has been growing interest in understanding the practical consequences of slipping into a mind wandering state. For example, when we mind wander, we now know that there is a systematic reduction in the extent to which we process external stimulus events at both the sensory and cognitive levels (e.g., Smallwood et al., 2008; O'Connell et al., 2009; Smilek et al., 2010; Kam et al., 2011; Hu et al., 2012), effects that can arise regardless of whether the events are task-related or not (e.g., Barron et al., 2011). In a corresponding manner, behavioral motor performance reliably shifts to a more automatic and/or degraded state (e.g., Schooler et al., 2004; Cheyne et al., 2006; Weissman et al., 2006; Carriere et al., 2008; Smallwood et al., 2008; Reichle et al., 2010), such that reaction times (RTs) tend to speed up and error rates are higher during mind wandering vs. on-task states (Smallwood et al., 2004; Franklin et al., 2011).

Yet despite such findings, our understanding of how mind wandering impacts motor behavior remains incomplete at best. Considered from a motor perspective, the range of potential mind wandering effects on behavioral control concerns more than just the speed and accuracy of response selection and the degree of response automaticity. In addition, the normal control of movement also involves the ability to adaptively monitor and adjust our motor outputs on a moment-to-moment basis as needed. Given that mind wandering attenuates the sensory and cognitive processing of external stimulus inputs, the goal of our study was to determine whether this may have a corresponding effect on our ability to dynamically adjust our motor behavior on-line in response to shifting, unpredictable environmental conditions.

In our first experiment we addressed the question using a canonical visuomotor tracking task that allowed us to measure the magnitude of continuous tracking error as a function of whether or not participants were in a mind wandering state. Tracking error did in fact increase during mind wandering. In our second experiment we examined whether this effect of mind wandering on behavior would generalize to a qualitatively distinct form of response monitoring and control—namely, feedback learning in the context of a time-estimation task. We again found behavioral evidence of the impact of mind wandering on the dynamic control of motor outputs, an effect that co-occurred with attenuations in direct, event-related potential (ERP) measures of performance monitoring processes in cortex.

In both experiments we relied on “experience sampling” as a means of determining the attention state of our participants over time (e.g., Schooler et al., 2011). Considered to be a “direct” measure of mind wandering, experience sampling relies on the fact that if prompted, we can reliably report on the content of our thoughts at any given moment and further, determine whether they center on the on-going task being performed (referred to as an “on-task” state), or alternatively, whether they have drifted off to other times, places, or issues (referred to as an “off-task” or “mind wandering” state) (for a review, see Gruberger et al., 2011). Although the act of reporting on one's thought state interferes with the content of consciousness itself (e.g., Filler and Giambra, 1973), by using the report to categorize a participant's attentional state in the 10–15 s immediately prior to the report, the methodology has been used to demonstrate reliable and replicable differences in neurocognitive functioning between “on-task” and “off-task” or “mind wandering” states (e.g., Smallwood et al., 2004; McKiernan et al., 2006; Mason et al., 2007; Smallwood et al., 2008; Christoff et al., 2009; Franklin et al., 2011; Kam et al., 2011; Stawarczyk et al., 2011; Kirschner et al., 2012). As such, in adopting this methodology here, our approach to defining attentional states aligned with widely-accepted norms in the field of mind wandering research.

## Experiment 1

In the first experiment, participants performed a visuomotor tracking task. They were stopped at unpredictable intervals and asked to report on whether their attention at that moment was “on-task” or whether they were “mind wandering.” To examine the influence of mind wandering on motor control, we compared the error in tracking performance between on-task and mind wandering states. Given that disruptive effects of mind wandering extend beyond perceptual and cognitive processes to response selection, we predicted there would be more errors during mind wandering relative to on-task states.

### Methods

#### Participants

Twenty-two participants completed the experiment in exchange for one course credit. They were all right handed, with no history of neurological problems and had normal or corrected-to-normal vision. Participants provided written informed consent to the experimental procedure. The Clinical Research Ethics Board at the University of British Columbia approved this study.

#### Task paradigm and procedures

**Figure 1:**
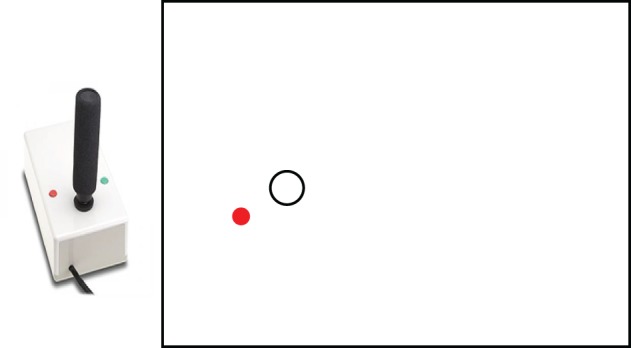
**Task paradigm of Experiment 1.** Participants were instructed to continuously track a moving target across the computer monitor using a joystick.

Participants performed a visuomotor tracking task (Boyd and Winstein, 2004; Boyd and Linsdell, 2009), in which they continuously tracked a target moving in sine-cosine waveform on a computer monitor by controlling the position of a cursor using a joystick. The target appeared as an open white circle and participant's movements were represented as a filled red dot on the monitor. The paradigm is shown in Figure 1. The task was to track the vertical path of the target with the joystick as accurately as possible. Joystick position sampling and stimuli presentation were both at 60 Hz, using custom software developed on the LabView platform (v. 7.1; National Instruments Co.).

There were 14 blocks of varying duration; lasting from 48 to 192 s. Each trial was 32 s long, tracking the target from left to right across a 17″ computer screen. Trials contained a 2 s baseline and 30 s of tracking a unique sine-cosine segment; each 30 s waveform was unique and could not be learned, thus participants were required to attend to visual stimuli in order to track accurately. The pattern of target movement was predefined and modified from Wulf and Schmidt's method (1997). Waveforms were generated using the polynomial equation with the following general form (cf. Wulf and Schmidt's, 1997), using randomly inserted coefficients ranging from −5 to 5:

$$f(x)=b_{0}+a_{1}\sin(x)+b_{1}\cos(x)+a_{2}\sin(2x)+b_{2}\cos(2x)+\;\cdots \;+a_{6}\sin(6x)+b_{6}\cos(6x).$$

Importantly, neither the target or participants' movements left a trail, thus participants could not visualize the entire target pattern. To control of waveform difficulty across participants, each practiced the same set of random waveforms.

Our primary behavioral measure was the changes in root mean squared error (RMSE), which reflects the overall tracking error in the kinematic pattern. It is the average difference between the target pattern and participant movements (cf. Boyd and Winstein, 2004). The RMSE is calculated as follows:

$$\text{RMSE}=\sum_{i=1}^{n}{\left\{{\left(x_{i}-T_{i}\right)}^{2}/n\right\}}^{1/2}$$

#### Task-related attention

To measure task-related attention, participants were instructed to report their “attentional state” at the end of each block. Specifically, they were asked to identify their state immediately prior to the block termination as either being “on-task” (fully attentive to task performance), or “mind wandering” (unattentive to the task) at the block's end. Importantly, participants were provided with descriptions and examples of these two attentional states prior to the testing session. “On-task” states were defined as when one's attention was firmly directed toward the task, whereas “mind wandering” states were described as when one is thinking about other things than just the task. Attentional reports were recorded at the conclusion of each block, and these reports were then used to sort behavioral data based on “on-task” vs. “mind wandering” states. As mentioned above, block duration was randomly varied between 48 and 192 s in order to minimize predictability of block completion and maximize variability of attentional state at the time of block completion. The duration of the task itself was approximately 30 min.

#### Statistical analysis

In terms of comparing on-task vs. mind wandering states, the periodicity of shifts in these attentional states tends to approximate 10–15 s (e.g., Sonuga-Barke and Castellanos, 2007; Christoff et al., 2009). We thus examined the movement data in the last 12 s prior to the subjective report of each attentional state prompted by the probes (cf. Smallwood et al., 2008; Kam et al., 2011). Specifically, we conducted paired-samples *t*-tests to compare the RMSEs by averaging together data in the 12 s preceding each of the two attentional states (on-task vs. mind wandering) report. Although we had no knowledge as to how long participants had actually been in a particular attentional state at the time a subjective report was given, our analyses were based on the assumption and recent evidence (Sonuga-Barke and Castellanos, 2007; Christoff et al., 2009) that the 12 s prior to each report would, on average, reliably capture the given attentional state.

### Results

#### Tracking performance

Participants completed 14 trial blocks, of which 43% were reported as “on-task” and 57% as “mind wandering”—a typical breakdown of attentional states (Smallwood et al., 2008; Kam et al., 2011). The motor tracking performance, indexed by the RMSE, was examined as a function of participants' attentional states. The RMSE preceding reports of mind wandering (*M* = 4.71, *SD* = 1.90) appeared to be much greater than those preceding on-task reports (*M* = 3.93, *SD* = 0.70). This was confirmed by a significant paired-samples t-test (*t*_(21)_ = −2.23, *p* = 0.03).

### Discussion

In Experiment 1, we found greater error in motor tracking just preceding reports of mind wandering relative to reports of on-task. This suggests that mind wandering does impair the precision at which we control our motor behavior on a moment-to-moment basis. Given the lack of external feedback on the participants' performance, however, it is unclear whether the increased tracking error during mind wandering was due to visual sensory attenuation *per se* (Kam et al., 2011), or whether mind wandering can also down-regulate behavioral/performance monitoring. We addressed this question in Experiment 2.

## Experiment 2

We recorded participants' EEG as they performed a simple time-estimation task during which they received trial-by-trial feedback on the accuracy of their responses and were occasionally asked to report their attention state at that moment as “on-task” or “mind wandering.” To determine the impact of mind wandering on performance monitoring, we measured the feedback error-related negativity (fERN) elicited by task feedback in the intervals immediately preceding “on-task” vs. “mind wandering” reports. In particular, the fERN is an endogenously-evoked ERP component that indexes the extent to which we are monitoring the accuracy of our responses, such that its amplitude positively covaries with the magnitude of behavioral assessment (Miltner et al., 1997; Holroyd and Krigolson, 2007; Krigolson et al., 2009). If mind wandering attenuates feedback monitoring, then it predicted that the fERN would be lower in amplitude during periods of mind wandering vs. on-task attentional states.

### Methods

#### Participants

Fifteen participants (9 females; *M* = 24.8 years, *SD* = 2.20) completed the experiment in exchange for $20 (Canadian dollars). They were all right handed, with no history of neurological problems and had normal or corrected-to-normal vision. Participants provided written informed consent to the experimental procedure. This study was approved by the Clinical Research Ethics Board at the University of British Columbia.

#### Stimuli and paradigm

We recorded EEG and behavioral data while participants performed a time-estimation task (cf. Miltner et al., 1997; Holroyd and Krigolson, 2007). On each trial, participants were required to estimate the duration of one second by pressing a button after an initial auditory cue. The cue was presented at 3000 Hz for 25 ms. Following the participant's estimate, a feedback stimulus was visually presented for 1000 ms at fixation to indicate the accuracy of the guess. After the offset of the feedback stimulus, a blank screen was presented for 400, 500, or 600 ms. Therefore, each trial lasted approximately between 2400 and 2600 ms (i.e., 2500 ms on average). A trial was considered correct if a participant's response occurred within a window of time centered around one second (±100 ms), and was considered incorrect otherwise. In order to maintain a global probability of approximately 0.5 for correct and incorrect feedback stimulus, the size of the response window decreased by 10 ms each time a participant was correct, and increased by 10 ms each time a participant was incorrect.

#### Behavioral measure

We determined the mean absolute change in response time following correct and error feedback as a function of participants' attentional states. That is, the absolute difference in time estimates between the current and previous trial was calculated in percentages for each participant (cf. Holroyd and Krigolson, 2007), separately for correct and error feedback during on-task and mind wandering states. This measure allows us to examine participants' sensitivity to their own behavioral performance as a function of attentional state.

#### Task-related attention

Attentional reports were recorded at the conclusion of each trial block, and these reports were then used to sort ERP data based on “on-task” vs. “mind wandering” states. The protocol for measuring task-related attention is identical to Experiment 1 with the following exceptions. The block duration itself was randomly varied between 30 and 90 s (i.e., 12–36 trials), and the duration of the task itself was approximately 65 min.

#### Electrophysiological recording and analysis

During the task, electroencephalograms (EEGs) were recorded from 32 active electrodes using a Biosemi Active-Two amplifier system. All EEG activity was recorded relative to two additional electrodes located over medial-parietal cortex (CMS/DRL), amplified with a gain of 0.5 and digitized on-line at a sampling rate of 256 samples per-second. To ensure proper eye fixation and allow for the correction and/or removal of events associated with eye movement artifacts, vertical and horizontal electrooculograms (EOGs) were also recorded—the vertical EOGs from an electrode inferior to the right eye, and the horizontal EOGs from two electrodes on the right and left outer canthus. Offline, computerized artifact rejection was used to eliminate trials during which detectable eye movements and blinks occurred. These eye artifacts were detected by identifying the minimum and maximum voltage values on all recorded EOG channels from −50 to 600 ms post visual feedback stimulus for each event epoch, and then removing the trial from subsequent signal averaging if that value exceeded 150 μV, a value calibrated to capture all blinks and eye movements exceeding approximately 1° of visual angle. For each participant, ERPs for each condition of interest were averaged into 3000 ms epochs, beginning 1500 ms before visual feedback stimulus onset. Subsequently, all ERPs were algebraically re-referenced to the average of the left and right mastoid signals, and filtered with a low-pass Gaussian filter (25.6 Hz half-amplitude cut-off) to eliminate any residual high-frequency artifacts in the waveforms. The resulting ERPs were used to generate grand-averaged waveforms.

#### Statistical analysis

Statistical quantification of ERP data was based on minimum peak and mean amplitude measures relative to a −200 to 0 ms pre-stimulus baseline. In particular, we derived “difference waves” for the on-task and mind wandering conditions by subtracting the correct feedback averaged waveforms from the incorrect feedback averaged waveforms for each attentional state and participant from electrode site FCz, where the fERN is typically maximal (e.g., Holroyd and Krigolson, 2007; Krigolson et al., 2009), as it was in our data. The fERN was then subsequently identified by an automated computer algorithm as the maximal negative voltage between 250 and 350 ms on the difference waveforms following feedback stimulus onset (see Holroyd and Krigolson, 2007) for more on this peak-picking methodology).

Here we compared both behavioral and ERP responses in the last 15 s prior to the subjective report of attentional state prompted by the probes. That is, the ERP waveforms for each condition of interest (correct vs. error) were based on averaging together the EEG epochs for the six trials preceding each of the two attentional states (on-task vs. mind wandering) report. We extended the analysis period to 15 s prior to each attentional report as an attempt to maximize the number of events to include in each waveform average while not extending the window back so far in time as to consistently capture the preceding attentional state or transition period between states.

### Results

#### Behavioral performance

**Figure 2:**
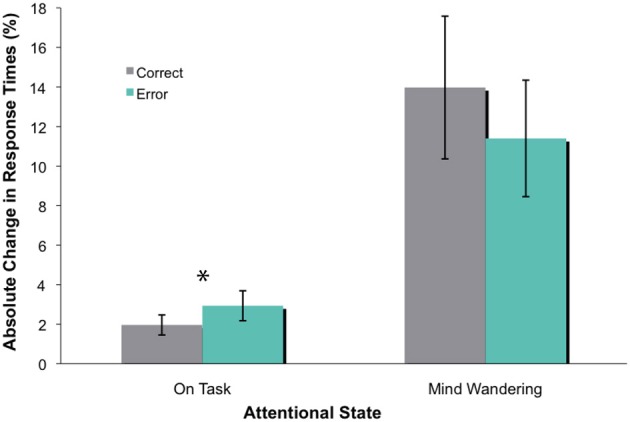
**The absolute change in time estimate (in percentage), with standard errors.** There was a significant difference between absolute change in time estimate following error and correct feedback during on-task states (as indicated by ^*^), but not mind wandering states.

Similar to Experiment 1, participants completed an average of 63 blocks of trials, of which 44% were reported as “on-task” and 56% as “mind wandering” (Smallwood et al., 2008; Kam et al., 2011). To examine how mind wandering affected behavioral performance, we conducted an omnibus ANOVA that had attentional state (on-task vs. mind wandering) and feedback valence (correct vs. error) as within-subject factors. The overall absolute change in time estimates and the variance of these time estimates during mind wandering periods appeared to be much greater than on-task periods, as shown in Figure 2. This data pattern was confirmed via a significant main effect of attentional state [*F*_(1, 14)_ = 39.51, *p* < 0.001]. The main effect of feedback valence was not significant [*F*_(1, 14)_ = 1.03, *p* = 0.328]. However, there was a significant attentional state X feedback valence interaction [*F*_(1, 14)_ = 8.95, *p* = 0.010]. Follow-up analyses revealed that the absolute change in time estimates following error feedback was significantly greater than that following correct feedback during on-task states [*t*_(1, 14)_ = −2.35, *p* = 0.034], but not during mind wandering states [*t*_(1, 14)_ = 1.93, *p* = 0.074]. While the adjustment in time estimates during mind wandering appears to be insensitive to feedback valence, this difference was nonetheless marginally significant. Along with the relatively small behavioral effect during on-task states, this set of finding makes it difficult to draw conclusions about the attentional effect on behavioral performance on this task.

#### Electrophysiology

Although the behavioral results showed evidence of decreased sensitivity to feedback during mind wandering, we wanted to first confirm normative mind wandering effects in our ERP findings, prior to assessing the fERN. In particular, the P3 elicited by target stimuli has been shown to reliably attenuate in amplitude immediately preceding reports of mind wandering relative to on-task (e.g., Smallwood et al., 2008; Kam et al., 2011). To confirm the reliability of our subjective reports, we thus wanted to determine that there was in fact a general attenuation of the P3 amplitude elicited by feedback signals immediately preceding mind wandering vs. on-task reports.

**Figure 3:**
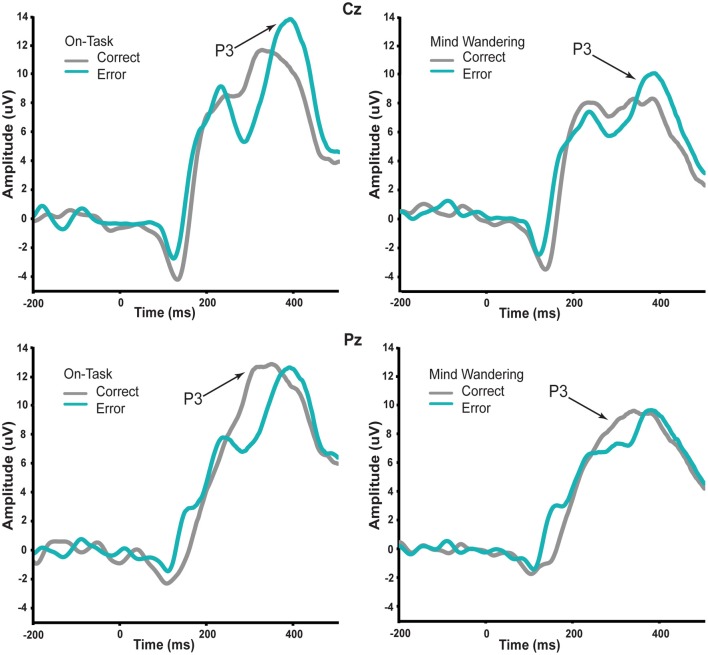
**P3 in response to correct and error feedback during on-task and mind wandering attentional states.** The amplitude of P3 at both Cz and Pz time-locked to the visual feedback stimulus was significantly reduced regardless of feedback valence during periods of mind wandering relative to periods of on-task.

Thus, we first conducted repeated-measures ANOVA on P3 with factors of attentional state (on-task vs. mind wandering), feedback valence (correct vs. incorrect), and electrodes (Cz and Pz) to establish the reliability of subjective reports of attentional state. For brevity, we only report effects associated with attentional state and feedback valence. The P3 elicited by the correct and error feedback as a function of attentional state is shown in Figure 3. This ERP component was measured at different time points between the two feedback stimulus types because it peaked at different time points for correct vs. error feedback, as can be seen in the figure. Mean amplitude measures were therefore taken across a 290–410 ms time window for correct feedback, and 330–450 ms time window for error feedback. We examined electrode sites Cz and Pz, where the P3 is typically maximal (e.g., Polich, 2007). There was a significant main effect of attentional state [*F*_(1, 14)_ = 12.06, *p* = 0.004] such that regardless of feedback valence, the P3 amplitude elicited by feedback signals was significantly greater immediately preceding on-task vs. mind wandering attentional reports. There was no main effect of feedback valence, nor an interaction between attentional state and feedback valence (*p* > 0.829). Importantly, this main effect of attentional state on P3 amplitude was consistent with the normative pattern for mind wandering (Smallwood et al., 2008; O'Connell et al., 2009; Kam et al., 2011).

**Figure 4:**
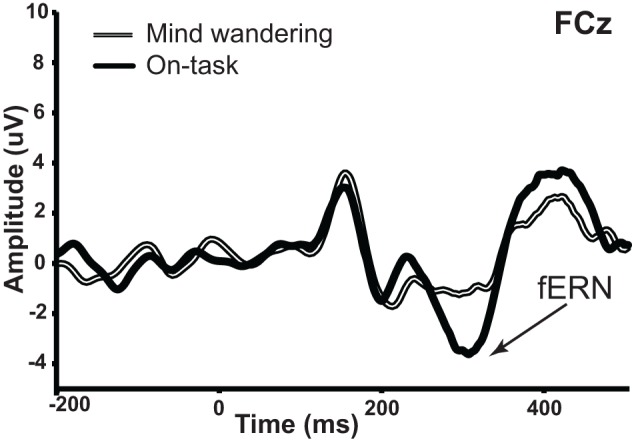
**fERN in difference waveforms (error—correct) as a function of on-task vs. mind wandering states.** The amplitude of fERN at FCz was significantly attenuated during periods of mind wandering relative to periods of on-task attention.

**Figure 5:**
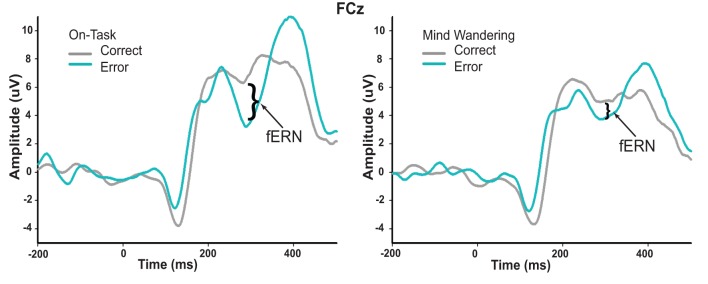
**Conditional waveforms of on-task and mind wandering attentional states in response to correct and error feedback.** The difference between correct and error feedback appears to be greater during on-task states relative to mind wandering states.

We then examined the impact of mind wandering on feedback processing, as measured via the fERN on the difference waveforms shown in Figure 4. The waveforms elicited by correct and error feedback stimulus as a function of attentional state are shown in Figure 5. As can be seen in Figure 4, the fERN appeared to be attenuated during mind wandering periods relative to on-task periods. To confirm this, two single-sample *t*-tests first confirmed the presence of a fERN in both the on-task [*t*_(14)_ = −5.43, *p* < 0.001, *d* = −2.90] and mind wandering [*t*_(14)_ = −3.75, *p* = 0.002, *d* = −2.00] conditions. Next, a comparison of the difference waveforms between on-task and mind wandering conditions revealed that the amplitude of the fERN was significantly reduced during mind wandering [*t*_(14)_= 2.22, *p* = 0.04, *d* = 0.61].

While definitive conclusions about the fERN can only be made with difference waveforms, we wanted to determine whether this fERN attenuation during mind wandering may be driven by a differential attentional modulation of the processing of correct and error feedback. As such, we compared the ERP waveforms of both correct and error feedback at FCz between on-task and mind wandering states, using the same individually-specified time windows as were used to identify the fERN in each individual's difference waveforms. In particular, we conducted repeated-measures ANOVAs with factors of attentional state (on-task vs. mind wandering), and feedback valence (correct vs. error). We found a significant interaction between attentional state and feedback valence [*F*_(1, 14)_ = 4.907, *p* = 0.044], suggesting that mind wandering was specifically attenuating fERN-related activity for correct feedback signals. This interpretation was confirmed via separate paired-samples *t*-tests, which revealed a significant main effect of attentional state in response to correct feedback [*t*_(14)_ = 2.691, *p* = 0.018], but not error feedback [*t*_(14)_ = 0.158, *p* = 0.877]. Specifically, while the processing of the correct feedback was significantly attenuated immediately preceding mind wandering (*M* = 6.16, SEM = 1.21) vs. on-task (*M* = 8.63, SEM = 1.31) attentional reports, the processing of error feedback did not significantly differ between mind wandering (*M* = 2.85, SEM = 1.09) and on-task (*M* = 3.04, SEM = 1.55) attentional states.

### Discussion

Using both behavioral and electrophysiological measures, Experiment 2 examined the question of whether mind wandering impacts the monitoring and adjustment of behavioral performance. We found decreased behavioral sensitivity accompanied by a reduced P3 to feedback stimulus during periods of mind wandering. Our data also revealed a reduced fERN during mind wandering compared to on-task attentional states. Consistent with the finding that correct trials appear to modulate the fERN amplitude (Holroyd et al., 2008), the reduced fERN was specifically driven by a significant mind wandering effect on correct, but not error, feedback.

## General discussion

The purpose of this study was to examine the effects of mind wandering on the online adjustment of behavior. Using a visuomotor tracking task in Experiment 1, we observed greater errors in tracking performance during periods of mind wandering. Using a time-estimation task in Experiment 2, we found reduced behavioral and neural sensitivity to performance feedback during mind wandering states, suggesting that the disruption in behavioral control could not be attributed to sensory attenuation *per se*. Extending previous research showing that mind wandering states decouple our attention from incoming sensory and cognitive stimuli (Smallwood et al., 2008; O'Connell et al., 2009; Kam et al., 2011), these results suggest mind wandering also disengages us from both monitoring and adjustment of our behavior.

That mind wandering was associated with increased error in a continuous tracking task is not surprising given mind wandering has been implicated in performance failures in vigilance tasks (Robertson et al., 1997; Smallwood et al., 2004) and response selection tasks (Schooler et al., 2004; Franklin et al., 2011). Interestingly, Boyd and Linsdell (2009) have implemented the motor tracking task over four practice sessions to induce motor sequence learning, and found that tracking performance at retention did improve as indexed by RMSE (Boyd and Linsdell, 2009). Given this finding, if mind wandering increases tracking error as we have shown in our study, this would not only lead to disruption in task performance and accordingly the learning of the sequence in the current testing session but it may also have a disruptive long term effect on the learning of motor skills over time.

If mind wandering is impacting behavioral feedback processing as measured via the fERN, how does this actually affect behavioral outputs? The fERN is time-locked to external signals of response accuracy, and is generated by a high-level error evaluation system that is tasked with performance optimization (Holroyd and Coles, 2002). As such, the fERN not only involves detecting the relative accuracy vs. inaccuracy of a response, but also reflects the extent to which we use that information for the modification of behavior (Krigolson et al., 2009). Given that mind wandering leads to transient reductions in the extent to which we process behavioral feedback signals, this suggests the functional consequences are two-fold. On the one hand, as our data confirm, the transient phases of mind wandering lead to direct disruption on the moment-to-moment adjustments in motor behavior. However, given that the cortical processes indexed by the fERN are associated with reinforcement learning (Holroyd and Coles, 2002), this would imply over time, mind wandering may also directly affect the trajectory or efficacy of motor learning itself. Together, findings from both experiments would suggest that the more we mind wander, the slower and less efficient motor learning may become.

Our report of a mind wandering effect on feedback processing manifest in the fERN raises the question to what extent might our findings be driven by these sensory and/or more general cognitive effects of mind wandering? In terms of possible visual sensory confounds, prior studies have found visual sensory attenuation for visual stimuli in the upper visual hemifield (Kam et al., 2011) but not for visual stimuli at fixation (Smallwood et al., 2008). As the visual feedback stimuli used in our study were at fixation, this suggests sensory attenuation is an unlikely explanation for our fERN results. Likewise, when we examined the P3 component in our study, we found attenuation in amplitude during mind wandering that was insensitive to the valence of feedback. In contrast, in the fERN, we found that the attenuation in amplitude during mind wandering was associated with a selective effect of mind wandering for correct feedback signals. This functional dissociation between the P3 and fERN findings thus suggests that the effect of mind wandering on the latter can not simply be ascribed to its effect on the former. Rather, it would appear that mind wandering can have a direct, independent influence on behavioral feedback processes in cortex.

Finally, given our results, it's also important to consider what our data are not showing. In particular, the fERN reflects an evaluation of one's preceding trial performance, based on delayed external feedback signaling whether or not behavior needs to be modified for improved performance. While the external feedback is typically presented in the form of a visual stimulus, the nature of this feedback and its implications in behavioral performance makes it qualitatively distinct from task-relevant sensory stimulus. In contrast, the response ERN is another error-related component that reflects the implicit aspect of response monitoring, whereby the internal evaluation of performance is based on the response itself (Gehring et al., 1993). While our findings suggest that mind wandering impacts the continuous adjustment of motor behavior in the absence of feedback as well as behavioral control associated with external feedback, whether it also affects the implicit evaluation of on-line performance as captured by the response ERN elicited by correct vs. incorrect responses remains to be directly investigated. If so, this would provide further support of the notion that mind wandering promotes response-independent thought.

Given our findings, an important issue concerns how if at all this relates to the attentional lapse literature. While mind wandering and attentional lapses capture a similar neurocognitive phenomenon, they do occur at very different temporal levels. In particular, mind wandering is a phenomenon that spans an extended period of time (i.e., fluctuations of 10–15 s) exceeding a given single event, whereas attentional lapses tend to occur during a much narrower time window capturing the lapse at a single event level. Several recent theoretical and empirical papers have supported and validated these two related models of attention (Dosenbach et al., 2008; Esterman et al., 2012). Specifically, at a theoretical level, Dosenbach and colleagues (2008) have suggested there are multiple controlling systems operating at multiple scales of time. Further, in terms of empirical evidence, the findings of Esterman and colleagues (2012) suggested the occurrence of two attentional states—one tied to the default mode network (reflective of mind wandering) that is more stable and less error prone in terms of behavioral measures, and a second one tied to the dorsal attention network (reflective of attentional lapses) that requires more effortful processing. That the effects of mind wandering appear to parallel effects of attentional lapses actually lends support to the notion that task-related attention (or mind wandering) and selective attention (or attentional lapses) may exert similar forms of top–down attentional control on other neurocognitive processes. In the case of attentional control of sensory response, it has been suggested that there are at least two distinct control systems operating in parallel—one associated with rapid shifts of selective visual attention (e.g., Mangun and Hillyard, 1991; Woldorff et al., 1997) and another one associated with slower fluctuations in task-related attention (O'Connell et al., 2009; Kam et al., 2011). In the case of behavioral control, that Weissman and colleagues have demonstrated that attentional lapses impair goal-directed behavior and are associated with reduced pre-stimulus activation in the anterior cingulate cortex (Weissman et al., 2006) and that we found impaired adjustment of behavioral control are consistent with the idea that varying attentional control systems appear to have similar impact on various neurocognitive processes. Taken together, mind wandering and attentional lapses do appear to be related conceptually, but future work needs to be done to disentangle the overlaying attentional influences linked to dissociable neural systems.

### Conflict of interest statement

The authors declare that the research was conducted in the absence of any commercial or financial relationships that could be construed as a potential conflict of interest.
